# Improved Neural Control of Movements Manifests in Expertise-Related Differences in Force Output and Brain Network Dynamics

**DOI:** 10.3389/fphys.2018.01540

**Published:** 2018-11-21

**Authors:** Christian Gölz, Claudia Voelcker-Rehage, Karin Mora, Eva-Maria Reuter, Ben Godde, Michael Dellnitz, Claus Reinsberger, Solveig Vieluf

**Affiliations:** ^1^Institute of Sports Medicine, Paderborn University, Paderborn, Germany; ^2^Institute of Human Movement Science and Health, Chemnitz University of Technology, Chemnitz, Germany; ^3^Department of Mathematics, Paderborn University, Paderborn, Germany; ^4^Centre for Sensorimotor Performance, School of Human Movement and Nutrition Sciences, The University of Queensland, Brisbane, QLD, Australia; ^5^Department of Psychology & Methods, Jacobs University Bremen, Bremen, Germany

**Keywords:** fine motor expertise, EEG, task-related brain activity, sensorimotor network, force control

## Abstract

It is well-established that expertise developed through continuous and deliberate practice has the potential to delay age-related decline in fine motor skills. However, less is known about the underlying mechanisms, that is, whether expertise leads to a higher performance level changing the initial status from which age-related decline starts or if expertise-related changes result in qualitatively different motor output and neural processing providing a resource of compensation for age-related changes. Thus, as a first step, this study aims at a better understanding of expertise-related changes in fine motor control with respect to force output and respective electrophysiological correlates. Here, using a multidimensional approach, we investigated fine motor control of experts and novices in precision mechanics during the execution of a dynamic force control task. On the level of force output, we analyzed precision, variability, and complexity. We further used dynamic mode decomposition (DMD) to analyze the electrophysiological correlates of force control to deduce brain network dynamics. Experts’ force output was more precise, less variable, and more complex. Task-related DMD mean mode magnitudes within the α-band at electrodes over sensorimotor relevant areas were reduced in experts, and lower DMD mean mode magnitudes related to the force output in novices. Our results provide evidence for expertise dependent central adaptions with distinct and more complex organization and decentralization of sensorimotor subsystems. Results from our multidimensional approach can be seen as a step forward in understanding expertise-related changes and exploiting their potential as resources for healthy aging.

## Introduction

The dexterous use of hands, including the precise modulation of fingertip forces, is required for many tasks of daily living. Normal hand functioning is realized by an elaborate and highly automated, and therefore efficient, system of neuromuscular control (Vaillancourt and Newell, 2002; Vieluf et al., 2015). The ability to precisely modulate fingertip force decreases with advancing age, starting in early middle adulthood and continuing through middle and old age (Lindberg et al., 2009; Diermayr et al., 2011). This decline may result in increasing demands on sensory, motor, and cognitive systems to maintain fine motor abilities during work and leisure activities. On the contrary, continuous and deliberate practice, leading to a domain specific expertise, can induce positive plasticity resulting in better performance and more efficient information transmission (Rosenbaum et al., 2006; Callan and Naito, 2014). Therefore, expertise might have a potential to postpone age-related decline in fine motor control (Vieluf et al., 2012). Up to now, however, the underlying processes that characterize expertise and expert performance are not sufficiently understood. Thus, it is necessary to characterize expertise-related changes in a suitable and standardized research context before exploiting its potential in terms of counteracting age-related decline or strengthening compensatory resources. In this study, we compared fine motor experts with novices in a force control task. Force control tasks require the precise adaptation of fingertip forces under visual control, as required in work routines of precision mechanics. To better understand expertise-related differences, we investigated behavioral markers of force control and electrophysiological correlates characterizing the neural control of movements in fine motor experts in comparison with novices within the working age range.

There is no unified definition of expertise, which already points to challenges in the investigation of this phenomenon. Experts are people who repeatedly, and not accidentally, perform excellently in a specific field or domain (Ericsson, 2006). This refers to a highly domain specific characteristic, skill, or knowledge that allows an expert to be distinguished from a novice (Ericsson and Lehmann, 1996; Ericsson, 2006). Based on this specificity, it is generally difficult to differentiate between experts and nonexperts in a scientific laboratory setting (Ericsson, 2014). The challenge in investigating the phenomenon-inherent properties and processes is to create a context that sufficiently reflects the specific field of expertise (Ericsson and Lehmann, 1996) and at the same time allows to define meaningful and standardized markers that differentiate between experts and nonexperts. Tasks requiring fine motor control provide such a research context in a suitable way. Fine motor tasks can be easily implemented in a laboratory setting and allow to characterize fine motor experts’ force control and its electrophysiological correlates. Until now, only few studies have examined fine motor experts in work-related contexts (Krampe and Ericsson, 1996; Law et al., 2004; Vieluf et al., 2012) such as the field of music (Krampe and Ericsson, 1996; Krampe et al., 2002). All studies showed a superior performance in fine motor control tasks in experts compared with novices. Using a force maintenance task, Vieluf et al. (2012) found experts’ performance to be less variable, more precise, and the time for force initiation to be shorter. Moreover, there are first indicators of a more complex or less regular performance output measured as center of pressure (CoP) fluctuations (Schmit et al., 2005; Stins et al., 2009) and for force maintenance (Vieluf et al., 2018) in experts. The higher complexity allows experts to be more adaptive to changes in task and environment indicating a higher motion automation and therefore fewer attentional control (Stins et al., 2009; Vieluf et al., 2018). Beyond behavioral improvements, based on the adaptation mechanisms inherent in the development of expertise, structural and functional changes occur in the brain (Rosenbaum et al., 2006). An enlargement of task-specific cortical areas in reaction to motor training was determined in monkeys by Nudo et al. (1996) and confirmed for humans in professional musicians (Elbert et al., 1995; Meister et al., 2005; Bangert and Schlaug, 2006) and Braille readers (Sterr et al., 1998). From a functional point of view, based on brain imaging studies, experts’ information processing seems more efficient (Callan and Naito, 2014; Debarnot et al., 2014) as reflected by more focused activation in task-relevant areas and higher suppression of task-irrelevant activities (Krings et al., 2000; Haslinger et al., 2004; Kim et al., 2008). On an electrophysiological level, increased efficiency was reflected by a reduction of cortical potentials in relevant areas in relation to the rest condition (Del Percio et al., 2008, 2009; Babiloni et al., 2010). Furthermore, neural efficiency was accompanied by a changed network characteristic. The functional organization of the expert’s brain was characterized by a stronger focus on communication between relevant areas and the simultaneous suppression of irrelevant connections (Bernardi et al., 2013; Binder et al., 2017) leading to a more efficient use and integration of information gained from several networks (Vieluf et al., 2018). Using a force maintenance task, Vieluf et al. (2018) point to the opposing relationship between expertise- and age-related processes, for which compensatory over activation of brain areas was reported earlier (Ward and Frackowiak, 2003; Reuter-Lorenz and Park, 2010) but could not clearly differentiate these processes.

In summary, these previous results indicate that experts perform better (i.e., more precise), with a less variable force output, and their behavioral performance is more complex, and while their information processing is characterized by increased neural efficiency. While these findings are based on investigations of simple or closed movements (Del Percio et al., 2008; Babiloni et al., 2011; Vieluf et al., 2018), phases before the execution of movements (Del Percio et al., 2011) or measurements during rest (Babiloni et al., 2010), it still remains open how expertise is characterized during the execution of a more complex domain-specific task with regard to its performance and information processing.

In extension of Vieluf et al. (2018), we aimed to identify expertise-related changes in the neural control of movements by examining experts of fine motor control in the execution of a complex domain-specific task. We proposed a dynamic force control task as a domain-specific task for precision mechanics. We used force and electrophysiological data from the Bremen-Hand-Study@Jacobs (Voelcker-Rehage et al., 2013) and selected markers that comprehensively describe force output and reflect the different electrophysiological characteristics of neural efficiency. In accordance with the studies mentioned above (Krampe and Ericsson, 1996; Krampe, 2002; Law et al., 2004; Vieluf et al., 2012), we expected fine motor experts to perform better in a force control task than novices in this domain. More specifically, in line with the studies of Stins et al. (2009) and Vieluf et al. (2012), we expected a more precise, less variable but more complex force output from the experts. Based on the study conducted by Brunton et al. (2016), for the analysis of the electrophysiological data, we used dynamic mode decomposition (DMD) to extract spatiotemporally coherent patterns of the captured electrical fields that represent the dynamic network characteristics of brain activity. Here, we expected to find indicators of increased neural efficiency, especially a changed network behavior, in the expert group. More precisely, owing to the change in information processing in experts as predicted by the neural efficiency hypothesis (Callan and Naito, 2014), we assumed a more focused activity in task-specific sensorimotor areas. We also expected neural efficiency to be reflected in a stronger activation of a task-specific sensorimotor network whose internal communication is more focused and thus more centered. Similar to our previous findings for a force maintenance task (Vieluf et al., 2018), we assumed this to be reflected in lower DMD values over sensorimotor relevant areas. Additionally, we explored the relation between electrophysiological markers of neural efficiency and force output markers.

## Materials and Methods

This work is based on the Bremen-Hand-Study@Jacobs (Voelcker-Rehage et al., 2013), which aimed to characterize age- and expertise-related differences in fine motor control in a sample of novices and experts throughout the working age-range. The data presented in this paper were collected during the fourth session of this study. In the previous sessions, participants underwent a series of behavioral tests including measures of somatosensory performance (Reuter et al., 2012) and force control (Vieluf et al., 2012, 2013a,b).

### Participants

Data from 47 participants were analyzed in this study. All participants had given their informed consent to the procedures before participating. Participants were recruited via diverse communication media (flyers, telephone calls, and newspaper announcements) and received a reimbursement of eight Euros per hour. The study was approved by the ethics committee of the German Psychological Society and was in accordance with the ethical standards laid down in the Declaration of Helsinki.

As mentioned above, participants were divided into fine motor experts (exp: *n* = 25; age = 50 ± 9 years; 13 females, MVC = 59.36 ± 21.94 N) and novices (nov: *n* = 22; age = 51 ± 9 years; 13 females, MVC = 52.16 ± 21.94 N) based on their occupation and years of experience in a job requiring fine motor skills. Novices were defined as people whose daily work routines were hardly influenced by fine motor skills such as service employees (i.e., consultants, office clerks, insurance agents, and vocational trainees in these occupations). The expert group was comprised of participants with more than 10 years of experience in a field with high demands for fine motor control, here precision mechanics (e.g., optician, dentists, goldsmiths, watchmakers). A 10-year inclusion criterion was chosen based on the study conducted by Ericsson and Smith (1991), and the daily use of hands in the work context was verified with a questionnaire on the daily use of hands (Trautmann et al., 2011). In addition, clinical manual dexterity was assessed by using the Purdue Pegboard test performed with the right hand and in accordance with the manual (Model 32020, Lafayette Instruments, Lafayette, IN, United States). A questionnaire on demographic status and health identified the participants as healthy and free of neurological restrictions and limiting injuries of upper extremities. All participants had normal or corrected to normal vision and hearing. All participants were right-handed, which was assessed by the Edinburgh Handedness Inventory (Oldfield, 1971). Each participant had conducted more than 200 trials of various force control tasks within experiments during the previous sessions of the Bremen-Hand-Study@Jacobs. Consequently, all participants were highly familiar with the setup and tasks.

### Experimental Procedure

**FIGURE 1:**
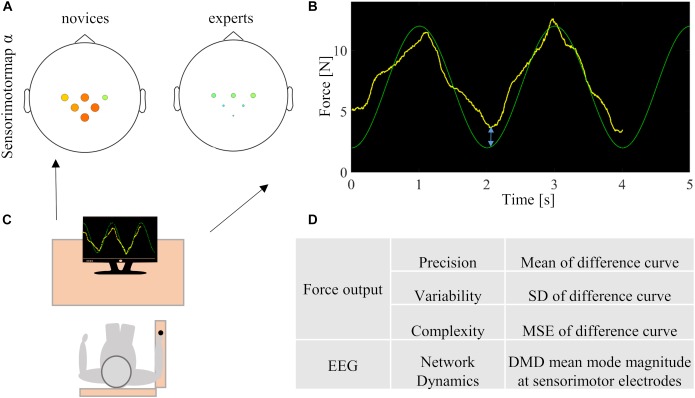
Illustration of the multidimensional approach. **(A)** DMD mean mode extraction at sensorimotor relevant electrodes. **(B)** Analysis of the difference of target and applied force, indicated by the blue arrow. **(C)** Experimental setup. **(D)** Overview of force output and electrophysiological markers (SD = standard deviation, MSE = multiscale entropy, DMD = dynamic mode decomposition).

Prior to the measurements, the maximum voluntary contraction (MVC) of each test subject was determined using the peak force achieved out of three maximum precision grip trials, 5 s each with 2 min of rest in between them (Vieluf et al., 2013b). After completion of the electroencephalogram (EEG) setup, we recorded 30 s of EEG at rest while participants sat on the chair with their eyes open. This resting state measure is required to relate the following analyses to the task and to normalize the differences between test persons. Afterward, participants performed a dynamic force control task, with their dominant right hand. Participants’ task was to match a curve that represented their applied force as precisely as possible to a target force presented on a screen at a distance of 80 cm (19″, frame rate 60 Hz). For this purpose, test persons sat on a chair with arms resting quietly on the armrests and thumbs and index fingers gripping a force transducer (Mini-40 Model, ATI Industrial Automation, Garner, NC, United States) fixated on the armrest. The target force changed over time in the form of a sinusoidal time curve, so that the test persons had to constantly adapt their force output with their thumbs and index fingers to the target value. The force time curve of the target curve averaged to 7 N (minimum: 2 N, maximum: 12 N) and was presented at a frequency of 0.5 Hz. Both the target and the force produced were displayed on the screen in front of the participants. The time axis (x-axis) covered 5 s. The force (y-axis) was presented in a range from 0 to 14 N. The target curve shifted from right to left, and the presentation of the produced force moved from left to right on the screen. In this setting, the participants always saw 1 s of the upcoming target force curve in advance and 4 s of the already exerted force and target curve (see Figures 1B,C). In order to ensure that the target force level could be reached as quickly as possible, the start of the target curve had been set in minimum (i.e., in 2 N). Participants performed seven trials of 30 s each. Familiarization was not carried out, as participants were already familiar with the setup and task from their previous visits. While performing the motor task, EEG was recorded.

### Data Recording

Grip force data were recorded with the force transducer with a sampling rate of 120 Hz with an amplitude resolution of 0.06 N using a customized LabView (National Instruments, Austin, TX, United States) program, which also provided online visual feedback on the screen.

Electroencephalogram data acquisition was done with a 32-electrode system with active electrodes (ActiveTwo, BioSemi, Amsterdam, Netherlands). The signal was recorded with a sampling rate of 2048 Hz and online band-pass filtered between 0.16 and 100 Hz. Electrodes were placed according to the 10–20 system (Jasper, 1958). In addition, the active common mode sense (CMS) electrode and the passive driven right leg (DRL) electrode were affixed next to Cz and used as reference and ground electrodes, respectively^1^. Vertical and horizontal eye movements as well as mastoid potentials were recorded with six facial electrodes designed for body-surface applications. Impedances were kept below 5 kOhm.

### Data Analysis

For data analysis, MATLAB 2016b (MathWorks, Natick, MA, United States) and the additional EEGLAB package 14.1 (Delorme and Makeig, 2004) were used.

#### Analysis of Force Output

For analysis of the force data, only the z-component of the recorded force vector was further analyzed. Initially, the force data were filtered offline with a fourth-order lowpass butterworth filter with a cutoff frequency of 30 Hz. In order to exclude the initiation phase, the first 2 s of the force-time signals were excluded from further analysis. The analysis finally included the absolute mean difference (arithmetic mean of the deviation from the target force), the magnitude of variability (standard deviation of the deviation from the target force), and the complexity [multiscale entropy (MSE)], as described in the study conducted by Costa et al. (2005) (see Figures 1B,D).

Multiscale entropy allows to assess force output in the context of underlying neurophysiological processes, which can be assumed to indicate adaptability (Costa et al., 2005; Vieluf et al., 2015). In addition, MSE is the calculation of sample entropy values over several scales based on a coarse-graining procedure of the signal. As with this procedure, multiple coarse-grained time series are constructed by averaging the data points within non-overlapping windows of increasing length; there are different frequency ranges inherent in these time series. This allows to focus on relevant scales informing about process dependent changes in the signals’ complexity (Morrison and Newell, 2012; Vieluf et al., 2015). To calculate the MSE, the vector length was fixed at 2, and the tolerance frame was 20% of the standard deviation of the signal (Costa et al., 2005; Vieluf et al., 2015). Based on the signal length and sampling rate, the entropy values of 60 scales were calculated. As an overall variable for the complexity of the signal, the arithmetic mean of the MSE values was determined over all scales (mean MSE). According to the inherent frequencies, the entropy values of functionally relevant scales were extracted based on the study conducted by Vieluf et al. (2015). These were scale 2 (inherent frequencies up to 30 Hz) representing the spectrum after filtering, scale 5 (inherent frequencies up to 12 Hz) representing the mechanisms of sensorimotor processing and physiological tremor (Elble and Randall, 1976; Vaillancourt and Newell, 2003), and scale 15 containing frequencies up to 4 Hz most relevant for sensorimotor processing (Slifkin et al., 2000; Vaillancourt and Newell, 2003).

To identify outliers due to incorrect test execution, trials whose absolute mean force levels were below or above 2.5 times the standard deviation with regard to the group mean were excluded from further analysis (Frank et al., 2006; Vieluf et al., 2017). Thus, in the novice group, trials that were less than 4.7 N or more than 8.6 N in relation to the mean force were rejected, and in the expert group, trials that were less than 5.6 N or more than 7.6 N in relation to the mean force were rejected. In total, eight trials were excluded (experts: seven trials, novices: one trial). This included all seven trials of an expert who was consequently excluded from all further analysis.

#### Analysis of Electrophysiological Data

Electroencephalogram data were resampled to 200 Hz according to the Nyquist theorem and cut based on trial onset and length. Next, the data were re-referenced to the linked mastoids and band-pass filtered using an FIR filter (low cut off: 4 Hz, high cut off: 30 Hz). Later, the recordings were checked semiautomatically for artifacts. Signal components whose difference of maximum and minimum exceeded 120 μV within a window of 20 ms were marked as artifact. This criterion was chosen based on the common EEG analysis software (see BrainVisionAnalyzer, Brain Products, 12.2.5 – 4). Furthermore, the signals and markings were visually inspected by the authors of the study. Wrongly detected artifacts were discarded, and undetected artifacts were added. For further analysis, the time signals were divided into segments with a length of 0.5 s (100 data points). Segments in which artifacts were detected were rejected and remained unconsidered. Owing to a technical artifact, all trials of one subject (expert) had to be excluded from further analysis. On average, nine segments per test person (nov: 10, exp: 8) were rejected. Linear detrending had been applied to cleaned data to eliminate voltage shifts. In addition, the signals were amplitude normalized to reduce its contribution in further analysis.

We used the exact DMD algorithm described in Brunton et al. (2016), which was first proposed by Tu et al. (2014). This algorithm allows observation of the expression of the signals over the scalp detected by the EEG electrodes in relation to each other and thus to draw a conclusion on the dynamic network behavior of the brain. Herewith, not only the entire network behavior but also the activity of certain (sub-)networks, such as the sensorimotor network, can be approximated. In addition, DMD approximates the relationship between two data series *X* and *X*′ in a time window. Time windows of 0.5 s length corresponding to 100 data points were analyzed. Linked spatial and temporal characteristics were approximated for each time window by a linear dynamical model given by *Y* =*Φ*exp(*Ωt*)*z*, where *Φ* is the DMD mode matrix, *Λ* is a diagonal matrix with DMD eigenvalues along the diagonal from which *Ω* = log(*Λ*)/*Δt* is obtained, *t* is time, *Δt* = 0.005*s*, and *z* is computed from the first data point *x* of *X*, that is, *x* =*Φz*. From the DMD eigenvalues, the oscillation frequency *f* was computed as *f* = |imag(*Ω*)/(2*π*)|. The mode matrix *Φ* represents the activation relationship between the electrodes for a particular frequency and indicates how much an electrode contributes to the dynamics of the network. The linear model illustrates how the spatial and temporal characteristics are linked. To increase the number of modes computed and thus the approximation accuracy, the delay embedding technique was applied, that is, data was stacked with a stacking depth of *h*. As a result of the error analysis on 100 randomly chosen windows of participant data, also described in Brunton et al. (2016), *h* = 2 was selected, as it revealed minimum error. The DMD analysis therefore was performed on an assembled data matrix, which stacked the first 99 data points on top of the last 99 data points. In addition, DMD mean mode magnitudes were calculated by averaging the mode magnitudes over all windows and associated with certain frequency ranges. A high DMD mode value indicates that the expression of the signal of a certain frequency recorded by each channel is high in relation to all other signals. By selecting the spatial distribution of the signal on seed electrodes, it is possible to draw conclusions about certain task-specific networks, such as the sensorimotor network. To characterize sensorimotor processes, we chose DMD mean modes at electrodes C3, C4, and Cz over sensorimotor regions. Furthermore, as Del Percio et al. (2011) and Binder et al. (2017) pointed out the importance of parietal areas in visuomotor tasks, we chose DMD mean modes at (centro-)parietal electrodes CP1, CP2, and Pz as seed electrodes and extracted the DMD mean modes, which are associated with the α- (8–12 Hz) and β- (12–30 Hz) frequency ranges. To obtain task-related values, the values of the rest condition were subtracted from the task condition in the same way as described in Brunton et al. (2016) (see Figures 1A,D). Finally, DMD mean modes of all valid trials were averaged per participant.

### Statistical Analysis

The statistical analysis included the mean value of all valid trials (maximum seven) of all participants excluding the outliers. After data processing, this comprised 23 experts and 22 novices. Analyses were performed using SPSS statistics 22 (IBM, Armonk, NY, United States). Normal distribution was tested using the Shapiro–Wilk test. Screening results were compared among groups using *t*-test for two independent samples and Mann–Whitney *U*-test in case of violation of normal distribution.

Multivariate analysis of covariance (MANCOVA) with the between factor group (2; experts, novices) controlling for age and MVC was conducted to determine significant differences between experts and novices on the level of force output. For the analysis of the electrophysiological data, we added the within subject factor electrode (6; C3, C4, Cz, CP1, CP2, Pz) to the model. In case of violations of sphericity, the Greenhouse–Geisser adjustment was used, and corrected degrees of freedom and p-values were reported. Significant interactions and main effects were followed by Bonferroni corrected pairwise comparisons. In addition to normal distribution, we checked for homogeneity of error variances and covariance. Levene’s test and Box’s test showed no violation here (both *p* > 0.05). As analysis of variance was shown to be a robust statistical procedure in case of violation of normal distribution, especially with almost the same group sizes and group sizes over 10, we decided not to choose nonparametric methods in case of violation (Box, 1954; Glass et al., 1972; Schmider et al., 2010). Following Cohen (1988) and as suggested by Lenhard and Lenhard (2014), we considered effect size of $\eta_{p}^{2}$ > 0.01 to 0.06 as small effects (equivalent to Cohen’s d of 0.2 to 0.4), $\eta_{p}^{2}$ > 0.06 to 0.14 as medium effects (equivalent to Cohen’s d of 0.5 to 0.7), and $\eta_{p}^{2}$ > 0.14 as large effects (equivalent to Cohen’s d of > 0.8).

To detect relationships between electrophysiological data and force output, variables of both levels were correlated using Pearson product-moment correlation, and in case of violation of the normal distribution Spearman rank correlation was used. False discovery rate (Benjamini and Hochberg, 1995) was used to correct the obtained p-values. As this analysis can be regarded as rather explorative, we report both uncorrected and corrected *p*-values. The correlation coefficients are judged according to the study conducted by Hopkins et al. (2009) with *r* > 0.1 to 0.3 indicating low, *r* > 0.3 to 0.5 indicating medium, *r* > 0.5 to 0.7 indicating strong, *r* > 0.7 to 0.9 indicating very strong, and *r* > 0.9 indicating perfect correlations.

## Results

### MVC and Pegboard Results

A Mann–Whitney *U*-test indicated that the frequency of hand use was higher in experts (median = 35) than in novices (median = 16, *U* = 12.5, *p* < 0.01). No group differences were detected in the MVC values (exp: median = 52.63 N, nov: median = 49.18 N, *U* = 196.00, *p* = 0.20) and the pegboard results [exp: *M* = 15.51, *SD* = 1.70, nov: *M* = 15.1, *SD* = 1.71, *t*(43) = 0.86, *p* = 0.40].

### Force Output Results

**FIGURE 2:**
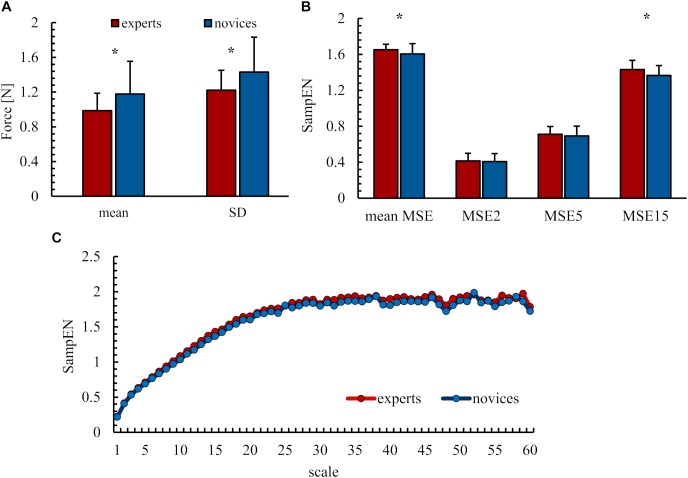
Behavioral results **(A)** mean difference (mean) and variability (SD). **(B)** Entropy values as mean over all scales and of chosen scales 2, 5, and 15. **(C)** MSE curve of entropy values over all scales. All values as group mean and standard deviation. ^∗^ Indicates significant differences.

Force output results are illustrated in Figure 2. Analyses revealed significant differences between experts and novices. Precision was higher, as indicated by lower mean differences, in the expert group (*M* = 0.97 N, *SD* = 0.20 N) than in the group of novices [*M* = 1.18 N, *SD* = 0.38 N; *F*(1,41) = 4.56, *p* = 0.04, $\eta_{p}^{2}$ = 0.10]. Experts’ force output was less variable (*M* = 1.23 N, *SD* = 0.40 N) than that of novices [*M* = 1.23 N, *SD* = 0.40 N, *F*(1,41) = 4.80, *p* = 0.03, $\eta_{p}^{2}$ = 0,11]. Differences in the complexity of the force output were found between the experimental groups. Experts’ MSE was higher overall [exp: *M* = 1.65, *SD* = 0.61, nov: *M* = 1.61, *SD* = 0.11, *F*(1,41) = 4.64, *p* = 0.04, $\eta_{p}^{2}$ = 0.10] and on scale 15 representing sensorimotor processes [exp: *M* = 1.43, *SD* = 0.10, nov: *M* = 1.36, *SD* = 0.61, *F*(1,41) = 4.29, *p* < 0.05, $\eta_{p}^{2}$ = 0.10], while no significant differences were observed for MSE scale 2 representing the spectrum after filtering [exp: *M* = 0.41, *SD* = 0.09, nov: *M* = 0.41, *SD* = 0.09, *F*(1,41) = 0.001, *p* = 0.91, $\eta_{p}^{2}$ = 0.00] and scale 5 representing the mechanisms of sensorimotor processing and physiological tremor [exp: *M* = 0.71, *SD* = 0.09, nov: *M* = 0.41, *SD* = 0.11, *F*(1,41) = 0.15, *p* = 0.67, $\eta_{p}^{2}$ = 0.004].

### Electrophysiological Results

**FIGURE 3:**
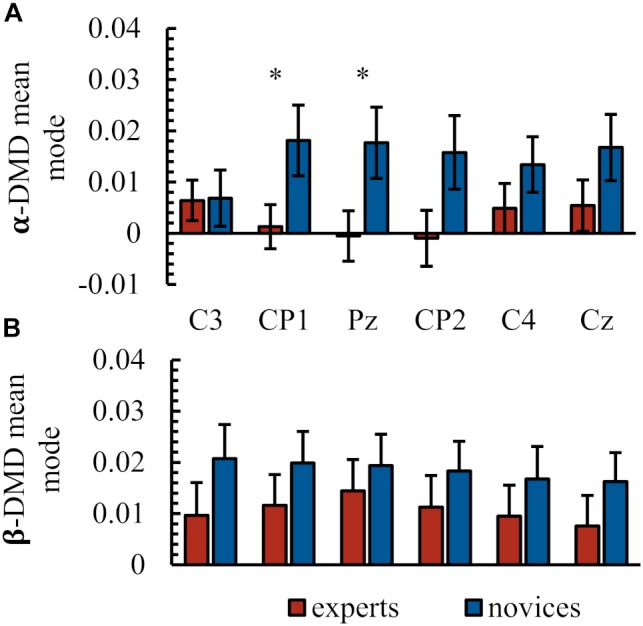
Electrophysiological results. Task-related DMD mean mode at the electrodes of interest C3, CP1, Pz, CP2, C4 in the α-frequency band **(A)** and in the β-frequency band **(B)**. All values as group mean and standard error. ^∗^ Indicates significant differences.

Figure 3 and Table 1 summarize the electrophysiological results. Task-related DMD mean modes of seed electrodes representing sensorimotor network activity were compared between groups. In the α-frequency band, statistical analysis revealed neither a significant main effect of group [*F*(1,41) = 2.99, *p* = 0.09, $\eta_{p}^{2}$ = 0.07] nor of electrode [Greenhouse–Geisser: *F*(3.38,138.71) = 0.47, *p* = 0.72, $\eta_{p}^{2}$ = 0.01]. A significant interaction between electrode and group was found in the task-related DMD mean modes in the α-frequency [Greenhouse–Geisser: *F*(3.38,138.71) = 3.72, *p* = 0.01, $\eta_{p}^{2}$ = 0.09]. *Post hoc* comparisons revealed significant differences between groups in task-related DMD mean mode magnitudes at CP1 (*p* = 0.05) and Pz (*p* = 0.04) and marginally significant differences at CP2 (*p* = 0.06) with lower values in the expert group (see Figure 2 and Table 1). No significant group differences were present in α- task-related DMD mean modes at central electrodes (C3: *p* = 0.88, C4: *p* = 0.21. Cz: *p* = 0.14). In the β-frequency band, statistical analysis revealed no significant main effect of group [*F*(1,41) = 1.54, *p* = 0.22, $\eta_{p}^{2}$ = 0.04] and a significant main effect of electrode [Greenhouse–Geisser: *F*(2.89,118.54) = 3.03, *p* = 0.03, $\eta_{p}^{2}$ = 0.07]. *Post hoc* comparisons revealed no significant differences between the electrodes here. No significant interaction between electrode and group was found in the task-related DMD mean modes in the β-frequency [Greenhouse–Geisser: *F*(2.89,118.54) = 1.05, *p* = 0.37, $\eta_{p}^{2}$ = 0.03].

**Table 1:** TR DMD mean mode magnitudes at electrodes of interest in the α-frequency and β-frequency.

	α-Band					
	CP1^∗^		CP2		Pz^∗^	
	Mean	*SD*	Mean	*SD*	Mean	*SD*
Experts	0.0013	0.0207	−0.0010	0.0262	−0.0005	0.0235
Novices	0.0181	0.0330	0.0158	0.0344	0.0177	0.0334

	**C3**		**C4**		**Cz**	
	**Mean**	***SD***	**Mean**	***SD***	**Mean**	***SD***

Experts	0.0064	0.0190	0.0049	0.0233	0.0054	0.0241
Novices	0.0068	0.0264	0.0134	0.0260	0.0168	0.0311

	**β-Band**					
	**CP1**		**CP2**		**Pz**	
	**Mean**	***SD***	**Mean**	***SD***	**Mean**	***SD***

Experts	0.0097	0.0307	0.0116	0.0288	0.0144	0.0293
Novices	0.0207	0.0319	0.0199	0.0295	0.0194	0.0292

	**C3**		**C4**		**Cz**	
	**Mean**	***SD***	**Mean**	***SD***	**Mean**	***SD***

Experts	0.0113	0.0295	0.0095	0.0290	0.0076	0.0284
Novices	0.0183	0.0278	0.0168	0.0304	0.0163	0.0271

*^∗^Indicates significant group difference.*

### Results of the Correlation Analysis

Significant positive correlations were found in the novice group for the correlation between task-related DMD mean mode in the α-frequency range at C3, CP1, and Pz and and mean difference [C3: *r* = 0.49, *p* = 0.02 (p_cor_ = 0.12); CP1: *r* = 0.52, *p* = 0.01 (p_cor_ = 0.12); Pz: *r* = 0.49, *p* = 0.02 (p_cor_ = 0.12)], SD [C3: *r* = 0.58, *p* < 0.01 (p_cor_ = 0.06); CP1: *r* = 0.62, *p* < 0.01 (p_cor_ = 0.06); Pz: *r* = 0.58, *p* < 0.01 (p_cor_ = 0.06)], and mean MSE [C3: *r* = -0.47, *p* = 0.03 (p_cor_ = 0.12); CP1: *r* = -0.46, *p* = 0.04 (p_cor_ = 0.14); Pz: *r* = -0.47, *p* = 0.03 (p_cor_ = 0.12)]. Furthermore, a significant positive correlation between task-related DMD mean mode in the β-frequency range at C4 and SD was found [*r* = 0.49, *p* = 0.02 (p_cor_ = 0.12)]. In the expert group, only β DMD mean mode at CP1 and MSE at scale 5 correlated significantly [*r* = -0.42, *p* = 0.05 (p_cor_ = 0.14)].

## Discussion

Based on the potential of domain specific expertise for delaying age-related decline in fine motor control, we aimed to identify expertise-related changes in the structure of the force output and the respective neural control processes. In an effort to meet the challenges in the investigation of expertise effects, we chose a dynamic force control experiment conducted in the context of the Bremen-Hand-Study@Jacobs (Voelcker-Rehage et al., 2013). The force control task resembles the dynamic grasping pattern frequently used by precision mechanics as part of their daily work routines. In summary, experts in this study showed a more precise and less variable force output. In addition, MSE analysis further revealed higher complexity of motor control output in experts. We further applied DMD to detect spatiotemporally coherent patterns within the EEG and found lower DMD values over sensorimotor relevant areas, which are indicators of different (i.e., more efficient) activities within the sensorimotor network for experts and novices in the α-frequency.

### Characteristics of Experts’ Force Output

In order to reveal differences in force control, the classical measures of motor performance, precision (absolute mean difference), and variability (standard deviation of the deviation from the target force) were applied. Similar to results gained in fine motor experts performing a finger tapping task (Krampe and Ericsson, 1996) or a task specific to the profession of surgeons (Law et al., 2004) and especially to findings from a static force control task using the same participants (Vieluf et al., 2012), experts were superior to novices in the dynamic force control task. This was reflected in a smaller deviation from the target force and a less variable performance, that is, the smaller amplitude in force fluctuations of the expert group.

In order to infer the underlying organization of the sensorimotor system, we further considered the complexity of the force output (MSE). Compared with novices, experts showed a more complex force output averaged over all scales (mean MSE) and particularly on the scale related to sensorimotor processes (MSE 15) but not on the scale related to tremor frequencies (MSE 5). Thus, extensive practice seems to alter neural processes of motor control but not general age-related characteristics. With this finding, we were able to, for the first time, describe the complexity of the force output in a dynamic force control task in the context of expertise and were thus able to extend the findings of a force maintenance task (Vieluf et al., 2018). Previous studies in healthy older adults and patients postulated the loss of complexity hypothesis (Vaillancourt and Newell, 2002). According to this hypothesis, a decline in complexity is present with advancing age and diseases (Lipsitz and Goldberger, 1992; Vaillancourt and Newell, 2002). The higher complexity found in experts in this work would follow the principle of this hypothesis in the opposite way. Similarly, studies on force maintenance (Vieluf et al., 2018) and on postural control (Schmit et al., 2005; Stins et al., 2009) found higher complexity of the CoP oscillation pattern in experts compared with novices. A higher complexity in experts could be an indicator for greater motion automation (Stins et al., 2009). In other words, experts may need less attention and therefore less mental resources to accomplish the task. This would correspond to a higher efficiency in movement control. Furthermore, the increased complexity in experts could indicate a greater movement flexibility, as postulated by Schmit et al. (2005). Overall, we were able to confirm a superior performance of the expert group using the classical measures, precision, and variability. More interestingly, by assessing the complexity of behavioral performance via MSE, we provide first indications of a changed organization of sensorimotor control in experts. Such expertise-specific reorganization might allow for more adaptability when performing tasks.

### Electrophysiological Markers of Experts’ Force Control

To investigate electrophysiological correlates of sensorimotor processes, we used DMD to capture spatiotemporal patterns at sensorimotor relevant electrodes. Experts showed lower DMD mode magnitudes with significant differences in the (centro-) parietal electrodes in the α-frequency band. As DMD modes reflect the relation between all (sensorimotor relevant and irrelevant) electrodes, a lower DMD mode in the relevant frequencies above the sensorimotor areas could indicate a more focused activity within a sensorimotor network. This would further suggest a high specialization of this network. Owing to the localization of the seed electrodes over sensorimotor relevant brain areas, the α-frequency could be interpreted here as motor related (Pineda, 2005). Consequently, the results at this frequency could be considered in the context of a more efficient translation of sensory information into motor information modulated by a stronger sensorimotor network (Pineda, 2005). The importance of integration and conversion of sensory information could be reflected in the rather parietally localized differences between experts and novices. This could further suggest a higher network efficiency of the sensorimotor network in the expert group, especially regarding the processing of sensory information, which could be reflected in higher motion automation as described above. The force control task required the sensorimotor integration of visual information with force output. Participants were strongly dependent on visual feedback but could also use feedforward control based on the 1 s target preview. These findings are consistent with the study conducted by Binder et al. (2017), who described a stronger sensorimotor functional network in experts during the execution of various visuomotor tasks and emphasized the importance of the parietal areas herewith. Del Percio et al. (2011) also illustrated the coupling of parietal regions during the preparatory phase in shooting. Thus, it is conceivable that experts more effectively integrate visuomotor information. We failed to find any expertise-related effects for the β-DMD mean modes. The participants in our study came to the lab and performed force control tasks for the fourth time so that all participants (experts and novices) might have partly automatized the execution of the task and thus differences in the β-band might be reduced. While the superior behavioral performance of experts suggests that force control tasks are sensitive to expertise effects, the task may not have completely represented the respective expertise context (i.e., force modulation requirements at work). This might have influenced these results. Consequently, it is possible that expertise effects were lower than they would have been in even more specific tasks. Finally, a high between-subject variability could also be observed on the electrophysiological level in both frequency bands, which suggests a high individuality of the sensorimotor network.

### Combined Reflection of Electrophysiological and Force Output Markers

The explorative correlation analysis revealed that for the novices, but not the experts, lower α-DMD mean modes at C3, CP1, and Pz were associated with the less variable and more precise force output. Furthermore, a lower mean MSE was associated with higher α-DMD mean modes at these electrodes in novices. In the β-band we further found that lower DMD mean modes at C4 were associated with a lower variability of the force output. In the expert group, there was only a negative correlation between the β-DMD mean mode at CP1 and the MSE of scale 5: the lower the β-DMD mean mode, the higher the MSE of scale 5. Speculatively stated, there could be a connection between the dynamic network characteristics (neural efficiency) and the performance level in the group of novices. Babiloni et al. (2011) found similar associations between the coupling of the electrodes in the α-band over sensorimotor relevant parietal areas and performance. Although force control is an expression of many different internal processes, a more efficient execution (lower variability and higher precision) could be partially reflected in altered brain activity patterns indicating neural efficiency. On the other hand, there are no results in the expert group that point to a simple relationship between force control and electrophysiological markers. Rather, this could indicate a more complex interaction of central and decentralized subsystems, which could also be reflected in a higher complexity of the force output, especially on the sensorimotor scale (MSE 15). This points to the importance of multidimensional approaches in the analysis and characterization of expert performance.

Taken together, the force output and electrophysiological data confirm that continuous and deliberate practice at work leads to domain specific plastic changes of the fine motor control system. Alterations of the neuromuscular control are opposing the commonly observed changes with aging, that is, increase in error and variability as well as loss of complexity. In addition, the interpretation of the electrophysiological findings is in line with the neural efficiency hypothesis that experts recruit smaller and more specific networks, opposing the changes of brain activity with increasing age. The dedifferentiation hypothesis states that with increasing age a loss of specificity occurs. Thus, structures and mechanisms that are specialized in young adults become less distinct or common to different functions in older age (Baltes and Lindenberger, 1997; Reuter-Lorenz and Park, 2010). The reversal effects on multiple levels suggest that continuous and deliberate practice has the potential to postpone or counteract age-related declines. These results might offer a foundation to design targeted interventions aiming to counteract age-related losses. Correlations between force output and electrophysiological markers were only present in the group of novices. Potentially, this indicates a more complex interaction between central and decentral systems in experts.

### Methodological Considerations

This study can only provide first insights into expertise-related processes of the neuromuscular system as it is a cross-sectional study. A longitudinal study would expand the findings here and help to gain knowledge of how expertise is impacted by its development and maintenance into older age to elucidate the power of expertise in the context of aging. The chosen laboratory context and task can be considered as a suitable context since group differences are present in the force control task but not in the pegboard test. We used fixed force levels instead of making the force requirements relative to the MVC of the test persons. This had the advantage of mapping different requirements of the everyday task context and thus created a research context more similar to the expertise context. Nevertheless, the relatively low force levels could have had the disadvantage of different strength requirements for the participants. Furthermore, it should be noted that the participants here had already participated in three force control experiments, which could have had an influence on our results. Moreover, in this work, a resting measurement with open eyes was chosen as a baseline for the EEG signal. Such a baseline generally has the disadvantage of a higher exposure of the signals to artifacts caused by eye movements and blinking, which were removed during pre-processing. However, visual stimuli may have had a possible influence on resting activity. Nevertheless, such a rest condition was used in this study to weaken the effect of a dominant visual (sub-) network, which we expected to be engaged in the task. Therewith, we aimed to ensure that the examined sensorimotor network characteristics reflected a higher relation to the task itself.

In addition to traditional markers on the level of force output, we used the nonlinear method to sample entropy on different time scales. A general drawback of such methods is the dependency of input parameters (e.g., vector size, tolerance frame). As we chose these parameters in line with previous studies (Costa et al., 2005; Vieluf et al., 2015), we assume that our choice is valid. The main limitation for the interpretation of the electrophysiological data however is the small number of electrodes (*n* = 32) and the restriction to signal space. Thereby, the interpretation of the decrease of DMD mean modes as a decoupling remains speculative, though consistent with the literature. Increasing the number of channels, MRI co-registration, and transferring the signals into source space would overcome the general low spatial resolution of EEG in order to sharpen the results especially with regard to the sensorimotor regions.

At last, it should be noted again that the correlation analysis is explorative and the interpretation is based on the uncorrected *p*-values. Therefore, interpretation of the correlation results should be done with caution.

## Conclusion

Here, we could confirm experts’ performance to be more precise, less variable, and more complex, pointing to a superior performance and changed organization of sensorimotor control. The latter idea is supported by the finding that complexity is higher for the MSE scale representing sensorimotor processing but not for tremor. Electrophysiological correlates of force control further indicate that information processing might be more efficient in experts compared with novices. However, only in novices, we found a directional relationship between network characteristics and force output. This points to the importance of examining expertise with comprehensive multidimensional approaches. In summary, this study extends the knowledge in the field of expertise. Understanding the changes related to continuous and deliberate practice provides important insights into the characteristics of a fully developed expertise. Considering these characteristics (i.e., neural efficiency, higher complexity) in connection with results from aging research suggests that expertise could be taken up as an opponent to age-related changes. Nevertheless, it still remains open whether expertise-related specificity and efficiency can be transferred to non-expertise tasks and whether expertise-related changes tend to favor reverse effects or the development of compensational resources for age-related decline. We suggest that further investigations are needed to understand how and to what extent age-related changes can be affected by continuous and deliberate practice. This study could provide a starting point here.

## Author Contributions

E-MR, CV-R, BG, and SV set up the experiments. CG, E-MR, CV-R, BG, and SV were involved in the conception of the work. E-MR and SV collected data. CG and KM analyzed data. All authors interpreted results, drafted parts of the work, approved the final version of the manuscript, and agreed to be accountable for all aspects of the work.

## Conflict of Interest Statement

The authors declare that the research was conducted in the absence of any commercial or financial relationships that could be construed as a potential conflict of interest.

## Footnotes

1 https://www.biosemi.com/faq/cms&drl.htm
